# Solvent/Detergent-Treated Plasma in the Management of Pediatric Patients Who Require Replacement of Multiple Coagulation Factors: An Open-Label, Multicenter, Post-marketing Study

**DOI:** 10.3389/fped.2020.00572

**Published:** 2020-09-17

**Authors:** Philip C. Spinella, Santiago Borasino, Jeffrey Alten

**Affiliations:** ^1^Division of Critical Care, Department of Pediatrics, Washington University at St. Louis, St. Louis, MO, United States; ^2^Cardiovascular Intensive Care Unit, Division of Pediatric Cardiology, University of Alabama at Birmingham, Birmingham, AL, United States

**Keywords:** bleeding, neonate, octaplas, pediatric, post-marketing, safety, surgery

## Abstract

**Background:** Octaplas is a solvent/detergent-treated, pooled plasma used for the management of preoperative or bleeding patients who require replacement of single or multiple coagulation factors. The aim of this post-marketing study was to collect real-world data on octaplas treatment in pediatric patients, with the primary focus being safety.

**Methods:** This was an open-label, multicenter, phase IV study conducted in patients <16 years old who required replacement of multiple coagulation factors due to liver dysfunction associated with coagulopathy and/or required cardiac surgery or liver surgery. Octaplas was administered intravenously based on ABO-group compatibility. The primary endpoints included the incidence of serious adverse events (SAEs), adverse drug reactions (ADRs), thrombotic events (TEs), thromboembolic events (TEEs) and hyperfibrinolytic events (HFEs).

**Results:** A total of 50 patients were enrolled (≤2 years old, *n* = 37; >2 years old, *n* = 13; female, *n* = 24) and 49 patients completed the study. Indications for the use of octaplas included planned cardiac surgery (*n* = 40, 80.0%), liver transplant surgery (*n* = 5, 10.0%) and liver dysfunction (*n* = 5, 10.0%). No ADRs, HFEs or treatment-related TEs and TEEs occurred during the study. Five patients had SAEs, one of which was fatal (iatrogenic injury). Other SAEs included hemorrhage, hypotension, hemorrhagic shock, coronary artery hemorrhage, intracardiac thrombus, supraventricular tachycardia, portal vein thrombosis and respiratory failure (1 each). None of the SAEs were considered to be related to octaplas.

**Conclusions:** Results of the present study support the use of octaplas in the management of preoperative or bleeding pediatric patients who require replacement of multiple plasma coagulation factors.

**Clinical Trial Registration:** ClinicalTrials.gov identifier: NCT02050841.

## Introduction

Correction of abnormal coagulation profiles in non-bleeding patients is the most common indication for plasma transfusion (1, 2). Clinical practice guidelines recommend plasma transfusion in children and neonates with an abnormal coagulation profile and clinically significant bleeding or prior to invasive procedures that have a high risk of significant bleeding (3).

Several types of plasma products are available for transfusion, including fresh frozen plasma (FFP), plasma frozen within 24 h (FP24) and solvent/detergent-treated plasma (SDP) (4, 5). FFP and FP24 units are collected from a single donor, which results in a significant variability in coagulation factor levels between individual units (6). In contrast, SDP is pooled from multiple units collected from different donors, resulting in significantly lower variability in coagulation factor content compared with FFP and FP24 (7–9). This difference in coagulation factors results in greater variability in the generation of thrombin with FFP and FP24 compared with SDP (6). Also, the solvent/detergent treatment during the manufacturing of SDP inactivates any lipid-enveloped viruses, such as human immunodeficiency virus, hepatitis B and C, and Zika virus that may be present in the donated plasma (10–12). Compared with FFP, SDP is practically cell free, and contains significantly lower residual platelet concentrations and negligible amounts of microparticles due to its manufacturing process (6).

Octaplas (Octapharma AG, Austria) is a blood group-specific SDP prepared from 630 to 1520 single-donor FFP units. It is also marketed as octaplasLG, octaplasma, omniplas and other trade names in various countries. In 2013 octaplas was approved in the USA, and is indicated for the replacement of multiple coagulation factors in patients with liver disease or who are undergoing cardiac surgery or liver transplant and is also indicated for therapeutic plasma exchange (TPE) in patients with thrombotic thrombocytopenic purpura (13). The aim of this post-marketing study was to collect real-world data on octaplas treatment in pediatric patients, with the primary focus being safety.

## Materials and Methods

### Design and Patients

This was an open-label, multicenter, post-marketing phase IV study that included pediatric patients aged <16 years who required replacement of multiple coagulation factors due to liver dysfunction associated with coagulopathy or required coagulation factors prophylaxis prior to cardiac surgery or liver surgery (ClinicalTrials.gov identifier: NCT02050841). Patients were identified for potential enrollment in the study by monitoring for plasma orders by the clinical team. Those who were deemed likely to require an intra-operative plasma infusion were also invited to participate, but were excluded from the analysis if they subsequently did not receive plasma (i.e., screening failure). The main exclusion criteria were: history of hypersensitivity reactions to blood or plasma-derived products or to any excipient of octaplas; homozygous congenital deficiency of protein S; immunoglobulin A deficiency with documented antibodies against immunoglobulin A; congenital factor deficiencies or platelet disorders; use of any plasma product other than octaplas within 72 h of the first octaplas infusion; extracorporeal membrane oxygenation at the time when plasma for the first infusion episode was ordered by the treating physician; expected transfusion of >40 mL/kg of all blood products in a 24-h period; TPE; and premature birth, defined as gestation <37 weeks. In addition, plasma replacement >72 h after the end of cardiac surgery was an exclusion criterion for patients who did not have coagulopathy due to liver dysfunction, i.e., a patient requiring substitution of coagulation factors, due to liver dysfunction, more than 72 h after surgery was eligible for enrollment.

The study consisted of a screening phase, pre-infusion assessments, a 72-h treatment period and a 72-h safety follow-up period. A final examination was conducted at the end of the safety follow-up period.

This study was carried out in accordance with the recommendations of the local Institutional Review Boards at participating institutions. Institutional Review Board approvals for the protocol have been obtained. All subjects gave written informed consent in accordance with the Declaration of Helsinki.

### Treatments

Octaplas was administered intravenously at the investigational site by study staff based on ABO-group compatibility. In urgent cases and in cases where the same blood group was not available, blood group AB was used.

An infusion episode was defined as administration of any amount of octaplas, with ≤60 min between two individual infusions. The exact dose, number of infusion episodes and total volume of octaplas depended on the clinical setting. Normal infusions were defined as all non-bypass priming infusions received by the study population.

The following concomitant therapies were permitted: intravenous calcium-gluconate in patients with hypocalcemia due to presumed citrate toxicity; intravenous antihistamines and glucocorticoids in patients with allergic reactions; and red blood cells, cryoprecipitate, and platelet transfusions, if medically indicated. In addition, patients were permitted to receive extracorporeal membrane oxygenation while on study if it was initiated after enrollment. Single donor plasma (e.g., FFP, FP24, thawed plasma, etc.) and TPE were prohibited.

### Assessments and Endpoints

The primary endpoints of this study included the incidence of serious adverse events (SAEs), adverse drug reactions (ADRs), thrombotic events, thromboembolic events (TEEs), and hyperfibrinolytic events. Primary endpoint events that occurred between the start of the first infusion episode and the final examination were recorded. ADRs or adverse events (AEs) were elicited by clinicians from patients or their legal guardians using non-leading questions per clinical trial protocol standards. For example, “how have you been since the last evaluation?”, which a parent/guardian would answer if their child or adolescent was incapable of understanding or responding to the question. AEs were coded using the Medical Dictionary for Regulatory Activities. Study investigators graded the severity, seriousness and causality of all AEs captured on the electronic case report form. The Independent Data Monitoring Committee conducted a quarterly review of the safety data, scrutinizing any ADRs or SAEs. Non-serious AEs that were not related to octaplas were not recorded. ADRs were defined as AEs for which a causal relationship to octaplas could not be ruled out.

Secondary endpoints included: changes in hemostatic parameters (international normalized ratio [INR], prothrombin time [PT], activated partial thromboplastin time [aPTT], thromboelastography [TEG]); volume (dose in mL/kg) of octaplas used per infusion episode; medically significant changes in vital signs (heart rate, respiratory rate, body temperature, arterial saturation, and blood pressure); and investigator's assessment of overall safety for each patient (excellent: octaplas was well-tolerated; moderate: ADRs that were easily resolved or not clinically significant; poor: ADRs requiring significant medical intervention). Abnormal hemostatic parameter values were those outside of the range of normal for patients requiring plasma infusions for bleeding indications, based on the investigator's clinical experience. These were classified as clinically significant if, in the opinion of the investigator, they suggested a disease and/or organ toxicity that was new or that worsened from baseline, or if they required additional active management. Any abnormal values that did not fulfill these criteria were classified by the investigator as not clinically significant.

Blood samples were drawn within 6 h before the start of the first infusion episode and between 30 and 60 min after each infusion episode. The following TEG parameters were recorded: reaction time, time from the end of R until the clot reaches 20 mm, slope between R and K, maximum amplitude of clot strength, and estimated percentage lysis at 30 min.

### Statistics

A total of 50 pediatric patients were to be recruited in this study to provide adequate safety information in a reasonable amount of time, as discussed and agreed by the study sponsor with the United States Food and Drug Administration.

The safety analysis set included all patients who received ≥1 infusion of octaplas. The full analysis set was defined according to the intent-to-treat principle and comprised all patients who were included in the safety population and had data on the primary endpoint variables. The per-protocol population included all patients who completed the infusion episodes and the final examination without major protocol deviations that could have affected the study outcomes.

All data collected were summarized and presented descriptively. Descriptive statistics, including the number of cases, mean and standard deviation (SD), median, maximum, and minimum were calculated for continuous variables. For categorical variables, frequencies and proportions were calculated. The denominator for these calculations was the total number of patients in the analysis population unless otherwise specified. No hypothesis testing was conducted because this was a descriptive post-marketing study and not designed to be a hypothesis-testing study. Pre- and post-infusion measurements were compared only descriptively. All statistical calculations were made using SAS (version 9.4).

## Results

### Study Population

Between July 2015 and December 2017, 50 patients treated at two study centers (Washington University at St Louis and the University of Alabama at Birmingham) were screened and enrolled in this study (Table 1). Of these, 37 patients were neonates or infants (aged ≤2 years) and 13 were children or adolescents (aged >2 years). Overall, 24 female and 26 male patients were included; the proportion of male patients was higher in the >2 years group (69.2%; Table 1). Replacement of multiple coagulation factors was required due to planned cardiac surgery (*n* = 40, 80.0%), orthotopic liver transplant (*n* = 5, 10.0%) and liver dysfunction associated with coagulopathy (*n* = 5, 10.0%). The latter included patients with a clinical diagnosis of sepsis-related coagulopathy in four children (8.0%) and hypoxic-ischemic encephalopathy in one (2.0%).

**Table 1:** Demographic data, full analysis set.

	**Infants ≤2 years *n* = 37**	**Children >2 years *n* = 13**	**Total *n* = 50**
Age (years)			
Mean ± SD	0.2 ± 0.6	7.2 ± 5.0	2.0 ± 4.0
Min, max	0, 2	3, 16	0, 16
Sex^a^			
Male	17 (45.9%)	9 (69.2%)	26 (52.0%)
Female	20 (54.1%)	4 (30.8%)	24 (48.0%)
Ethnicity^a^			
Hispanic or Latino	1 (2.7%)	0	1 (2.0%)
Not Hispanic or Latino	36 (97.3%)	13 (100.0%)	49 (98.0%)
Race^a^			
White	28 (75.7%)	6 (46.2%)	34 (68.0%)
Black or African American	8 (21.6%)	6 (46.2%)	14 (28.0%)
Asian	1 (2.7%)	0	1 (2.0%)
Other	0	1 (7.7%)	1 (2.0%)
ABO Blood Group^a^			
A	13 (35.1%)	3 (23.1%)	16 (32.0%)
B	6 (16.2%)	3 (23.1%)	9 (18.0%)
AB	3 (8.1%)	1 (7.7%)	4 (8.0%)
O	15 (40.5%)	6 (46.2%)	21 (42.0%)
Height (cm)			
Mean ± SD	63.1 ± 10.2	120.9 ± 28.3	78.1 ± 30.4
Min, max	46, 92	96, 171	46, 171
Weight (kg)			
Mean ± SD	6.66 ± 2.7	33.58 ± 36.1	13.66 ± 21.6
Min, max	3.4, 14.0	12.7, 144.0	3.4, 144.0
BMI (kg/m^2^)			
Mean ± SD	16.15 ± 2.9	18.98 ± 9.5	16.88 ± 5.4
Min, max	11.5, 26.5	11.7, 49.2	11.5, 49.2
Indication for replacement of multiple coagulation factors^a^			
Cardiac surgery	31 (83.8%)	9 (69.2%)	40 (80.0%)
Liver transplant surgery	3 (8.1%)	2 (15.4%)	5 (10.0%)
Liver dysfunction			
Sepsis-related coagulopathy	2 (5.4%)	2 (15.4%)	4 (8.0%)
Coagulopathy associated with HIE	1 (2.7%)	0	1 (2.0%)
Type of surgery^a^			
Cardiac	31 (83.8%)	9 (69.2%)	40 (80.0%)
Orthotopic liver transplant	3 (8.1%)	2 (15.4%)	5 (10.0%)
None	3 (8.1%)	2 (15.4%)	5 (10.0%)
ECMO during surgery^a^			
Yes	0	1 (7.7%)	1 (2.0%)
No	36 (97.3%)	12 (92.3%)	48 (96.0%)
CPB during surgery^a^			
Yes	31 (83.8%)	9 (69.2%)	40 (80.0%)
No	5 (13.5%)	4 (30.8%)	9 (18.0%)
Concomitant medical conditions^a^			
Congenital, familial and genetic	30 (81.1%)	8 (61.5%)	38 (76.0%)
Ventricular septal defect	13 (35.1%)	4 (30.8%)	17 (34.0%)
Tetralogy of Fallot	16 (43.2%)	0	16 (32.0%)
Atrial septal defect	9 (24.3%)	2 (15.4%)	11 (22.0%)
Cardiac	18 (48.6%)	8 (61.5%)	26 (52.0%)
Respiratory, thoracic, and mediastinal	16 (43.2%)	7 (53.8%)	23 (46.0%)
Infections and infestations	8 (21.6%)	5 (38.5%)	13 (26.0%)
Gastrointestinal	5 (13.5%)	5 (38.5%)	10 (20.0%)

a *Categorial data are presented as n (%)*.

*BMI, body mass index; CPB, cardiopulmonary bypass; ECMO, extracorporeal membrane oxygenation; HIE, hypoxic-ischemic encephalopathy; SD, standard deviation*.

In total, 49 patients (98.0%) completed the study. One patient (2.0%) was withdrawn from the study prematurely due to the use of a prohibited treatment (FFP). The supply of octaplas at the study site was not sufficient to meet the transfusion requirements of this patient and FFP had to be used. Therefore, 50 patients were included in the safety and full analysis sets, while 49 patients were included in the per protocol population.

### Extent of Exposure

All 50 patients included in the study had ≥1 infusion episode, while 10 patients had two infusion episodes, five patients had three infusion episodes and one patient had six infusion episodes. The mean number of infusions did not appear to vary greatly between age groups (2 in patients aged ≤2 years and 4 in those aged >2 years, Table 2). The mean octaplas dose and median infusion dose (normal infusions) are shown for infusion episodes 1 to 3 in Table 2.

**Table 2:** Exposure to octaplas infusions, safety analysis set.

	**Infants ≤2 years *n* = 37**	**Children >2 years *n* = 13**	**Total *n* = 50**
Total number of infusions^a^	2.3 ± 0.9	3.8 ± 3.1	2.7 ± 1.8
Octaplas dose administered (mL/kg)^a^			
Infusion episode 1	33.9 ± 21.1 (*n* = 37)	12.8 ± 5.8 (*n* = 13)	28.4 ± 20.6 (*n* = 50)
Infusion episode 2	15.3 ± 8.9 (*n* = 4)	29.9 ± 30.6 (*n* = 6)	24.1 ± 24.6 (*n* = 10)
Infusion episode 3	11.2 ± 3.5 (*n* = 3)	13.9 ± 0.9 (*n* = 2)	12.3 ± 2.9 (*n* = 5)
Dose of normal infusions (mL/kg)^b^^,^^c^			
Infusion episode 1	12.9 [4–69] (*n* = 12)	11.7 [3–26] (*n* = 8)	11.7 [3–69] (*n* = 20)
Infusion episode 2	14.0 [6–27] (*n* = 4)	20.0 [7–90] (*n* = 6)	14.2 [6–90] (*n* = 10)
Infusion episode 3	10.4 [8–15] (*n* = 3)	13.9 [13–15] (*n* = 2)	13.2 [8–15] (*n* = 5)

a *Data are presented as mean ± SD*.

b *Data are presented as median [range]*.

c *Normal infusions are those excluding octaplas use for priming the circuit for cardiac bypass*.

For infusion episodes 1 and 2, the median infusion rates for normal infusions (excluding octaplas use for priming the circuit for cardiac bypass) was 0.2 mL/kg/min and for infusion episode 3 it was 0.1 mL/kg/min; infusion rates appeared similar between age groups although no statistical comparison was made (data not shown). In the 28 patients who had bypass priming (all aged ≤2 years), the median dose administered was 19.1 (6–38) mL/kg and the median infusion rate was 143.2 mL/kg/min.

### Safety

No ADRs were reported during the study. Overall, five patients (10.0%) had a total of nine SAEs (Table 3), three patients aged ≤2 years and two patients aged >2 years. Of these patients, four had congenital heart disease and were undergoing cardiac surgery, while one had liver disease and was undergoing liver transplant surgery. The proportions of male and female patients who had SAEs were 11.5 and 8.3%, respectively. Of the five patients who had SAEs during this study, four had a total of five SAEs that were considered severe, while one patient had four moderate-intensity SAEs. One patient had an SAE that led to death (injury to the coronary artery). All of the SAEs observed were considered by each site's principal investigator to be expected AEs that were related to the patients' underlying disease or surgical intervention. Therefore, none of the SAEs were considered related to the study medication or led to a change in its dose or withdrawal. There were no clinically significant changes in vital signs between pre- and post-infusion, other than those which may be expected in a patient undergoing cardiac surgery [e.g., hypertension is a frequent occurrence postoperatively in cardiovascular interventions (14)].

**Table 3:** Serious adverse events, safety analysis set.

	**Infants** **≤2 years** ***n*** **=** **37**		**Children** **>2 years** ***n*** **=** **13**		**Total** ***n*** **=** **50**	
	**SAEs *n***	**Patients^a^**	**SAEs *n***	**Patients^a^**	**SAEs *n***	**Patients^a^**
Any	3	3 (8.1%)	6	2 (15.4%)	9	5 (10.0%)
Hemorrhage	1	1 (2.7%)	0	0	1	1 (2.0%)
Hypotension	0	0	1	1 (7.7%)	1	1 (2.0%)
Hemorrhagic shock	1	1 (2.7%)	0	0	1	1 (2.0%)
Coronary artery hemorrhage	0	0	1	1 (7.7%)	1	1 (2.0%)
Intracardiac thrombus	0	0	1	1 (7.7%)	1	1 (2.0%)
Supraventricular tachycardia	0	0	1	1 (7.7%)	1	1 (2.0%)
Portal vein thrombosis	1	1 (2.7%)	0	0	1	1 (2.0%)
Iatrogenic injury	0	0	1	1 (7.7%)	1	1 (2.0%)
Respiratory failure	0	0	1	1 (7.7%)	1	1 (2.0%)

a *n (%) for categorical data*.

*None of the SAEs was considered to be treatment-related*.

*SAE, serious adverse event*.

No hyperfibrinolytic events were reported during the study. There were two thrombotic events or TEEs reported in two patients (4%). One patient aged ≤2 years and who was undergoing a liver transplant had portal vein thrombosis. One patient aged >2 years who had congenital heart disease and was undergoing cardiac surgery had an intracardiac thrombus. Neither of the TEEs was considered to be treatment-related. For all 50 patients, the investigator assessment of safety was “excellent.”

### Treatment Response

Only three clinically significant abnormal hemostatic parameters were observed pre- or post-transfusion during the first infusion episode. One patient (2.0%) each showed a clinically significant elevated INR pre- and post-infusion, and one patient (2.0%) showed a clinically significant elevated aPTT value post-infusion.

As would be expected in this population, many patients showed either no change in hemostatic parameters or changes within the range of normal to non-clinically significant abnormal, after the first infusion episode (Table 4). Following the first infusion, the majority of patients had non-clinically significant abnormal values post-infusion for INR (*n* = 36, 72.0%) and PT (*n* = 38, 76.0%), while one-half of patients also had non-clinically significant abnormal maximum amplitude of clot strength TEG values post-infusion (*n* = 25, 50.0%). On the other hand, the proportion of patients with non-clinically significant abnormal values was numerically lower post-infusion than pre-infusion for the following TEG parameters: reaction time (56.0 to 34.0%), time from the end of R until the clot reaches 20 mm (46.0 to 32.0%), slope between R and K (46.0 to 30.0%), and estimated percentage lysis at 30 min (2.0 to 0%). There was a concomitant numerical increase in the proportion of patients with normal values for each of these TEG parameters, from 32.0 to 58.0% (reaction time), 42.0 to 60.0% (time from the end of R until the clot reaches 20 mm), 42.0 to 62.0% (slope between R and K), and 86.0 to 92.0% (estimated percentage lysis at 30 min).

**Table 4:** Patients with normal or abnormal hemostatic parameters before and after the first infusion episode.

**Parameter^a^**	**Pre-infusion**	**Post-infusion**
INR	*n* = 47	*n* = 49
Normal	33 (66.0%)	12 (24.0%)
Abnormal NCS	13 (26.0%)	36 (72.0%)
PT, sec	*n* = 47	*n* = 49
Normal	27 (54.0%)	11 (22.0%)
Abnormal NCS	20 (40.0%)	38 (76.0%)
aPTT, sec	*n* = 47	*n* = 48
Normal	27 (54.0%)	24 (48.0%)
Abnormal NCS	19 (38.0%)	23 (46.0%)
TEG – R-time	*n* = 44	*n* = 46
Normal	16 (32.0%)	29 (58.0%)
Abnormal NCS	28 (56.0%)	17 (34.0%)
TEG – K-time	*n* = 44	*n* = 46
Normal	21 (42.0%)	30 (60.0%)
Abnormal NCS	23 (46.0%)	16 (32.0%)
TEG – alpha angle	*n* = 44	*n* = 46
Normal	21 (42.0%)	31 (62.0%)
Abnormal NCS	23 (46.0%)	15 (30.0%)
TEG – MA	*n* = 44	*n* = 46
Normal	32 (64.0%)	21 (42.0%)
Abnormal NCS	12 (24.0%)	25 (50.0%)
TEG – EPL30	*n* = 44	*n* = 46
Normal	43 (86.0%)	46 (92.0%)
Abnormal NCS	1 (2.0%)	0

a *Data are presented as n (%)*.

*aPTT, activated partial thromboplastin time; EPL30, estimated percentage lysis at 30 min; INR, international normalized ratio; K-time, time from end of R until the clot reaches 20 mm; MA, maximum amplitude of clot strength; NCS, not clinically significant; PT, prothrombin time; R-time, reaction time; TEG, thromboelastography*.

## Discussion

The results of this open-label, multicenter, post-marketing study provide clinical evidence supporting the use of octaplas in critically ill pediatric patients who require replacement of multiple coagulation factors due to major surgery or acquired deficiencies associated with liver dysfunction. No ADRs, hyperfibrinolytic events or treatment-related thrombotic events and TEEs occurred during the study. Five patients had SAEs, one of which was fatal (iatrogenic injury). None of the SAEs were considered to be related to octaplas. Treatment response appeared favorable in the majority of patients, with TEG values that were either stable pre- vs. post-infusion or that changed from abnormal (non-clinically significant) to normal pre- vs. post-infusion for most TEG parameters.

While there were few unexpected clinically significant changes in the hemostatic parameters, there were several parameters (e.g., INR, PT, aPTT) for which patients experienced increased values post- vs. pre-infusion. As a result, there was a numerical increase in the proportion of patients with non-clinically significant abnormal hemostasis values after the first octaplas infusion vs. pre-infusion. This may have occurred for two possible reasons: first, patients who were bleeding may have continued to consume coagulation factors and platelets while receiving octaplas, and secondly, most of the patients in this study (80%) underwent surgery with cardiopulmonary bypass and the anticoagulation required may have affected post-transfusion hemostasis parameters.

The results of this study add further evidence to the use of octaplas in the pediatric population, which was previously examined in a retrospective study (15). In that study, 41 critically ill neonates received a mean ± SD dose of 18.4 ± 13.2 mL/kg of octaplas and 15 pediatric patients with severe liver disease received 38.0 ± 41.5 mL/kg of octaplas either before or after liver transplant (15). Among the neonatal patients, 31 (75.6%) received octaplas because they were considered to be at risk of bleeding due to coagulopathy, eight (19.5%) because they had clinical hemorrhage due to coagulopathy and two patients had neither hemorrhage nor coagulopathy. Among the pediatric patients, clinical conditions included autoimmune hepatitis, cystic fibrosis, and hepatoblastoma in two patients each and liver injury, portal hypertension and bleeding, and leishmaniasis in one patient each. Following transfusion, significant reductions in mean aPTT and PT, and an increase in fibrinogen were observed in neonates, while increase in platelets was not statistically significant. Significant changes were also observed in pediatric patients, including reductions in aPTT and PT, while changes in fibrinogen and platelets were not statistically significant, and no clinical AEs associated with octaplas were observed in either group (15). In another retrospective study of critically ill pediatric patients undergoing TPE (*n* = 35), normalization of hemostatic parameters with SDP with a low incidence of AEs (1.2%) was reported (16).

In a report from a single hospital, 29 pediatric patients (mean age: 6 years; range: 1 day to 15 years) received a median of 10.0 mL/kg of octaplas (range: 6.3 to 21.1 mL/kg). Indications for transfusion included priming of cardiopulmonary bypass machine in 15 infusion episodes (29.4%), transplant-associated thrombotic microangiopathy in 14 episodes (27.5%), and active bleeding in 11 episodes (21.6%). The remaining transfusions were performed due to prolonged PT and/or aPTT with no further details provided (9.8%) and prior to a procedure (9.8%). Mean PT decreased from 26 to 19 s and mean aPTT decreased from 65 to 52 s (17). No adverse reactions were observed (17).

Octaplas was compared with FFP in a retrospective analysis that included 105 pediatric patients aged <2 years who were undergoing tetralogy of Fallot repair (18). Most patients received octaplas and FFP transfusions in the operating room after cardiopulmonary bypass (93.8 and 85.0%, respectively). Immediately after surgery, INR and aPTT were significantly lower in patients who received octaplas than in those who received FFP, and the proportion of patients who received additional plasma transfusions was significantly higher in the FFP group compared with the octaplas group. Furthermore, the rate of postoperative infections requiring the use of antibiotics was significantly higher in the FFP group compared with the octaplas group, and no transfusion reactions were observed in either group (18). In a secondary analysis of a prospective, observational study that included 443 critically ill children who received plasma transfusions, FFP and SDP were associated with similar reductions in INR (*p* = 0.80). However, intensive care unit mortality was significantly lower with SDP compared with FFP (14.5 vs. 29.1%, *p* = 0.02) (4).

It is possible that the results obtained in the comparative studies described above is due, in part, to the greater uniformity of coagulation factor content of individual octaplas units compared with FFP units (6, 7). Differences in coagulation factor content are particularly important in patients who receive transfusions of only one or very few units, such as children and neonates. However, prospective comparative studies are required to confirm the safety or efficacy advantages of octaplas observed in these studies.

Our study had a number of limitations. Firstly, the study included a relatively small number of patients and had a short observation period. Also, no comparator group was included in the study. As a result, it is difficult to judge the clinical relevance of the treatment responses observed in our study. In contrast with the previous studies of octaplas in children, which only assessed INR/PT/aPTT/antithrombin III/activated clotting times, we also assessed treatment response with various TEG parameters. Since this study was designed to evaluate the safety of octaplas treatment in pediatric patients, the efficacy outcomes were presented descriptively without assessment of the relationship between octaplas treatment and TEG measurements. Lastly, the influence of patients' ongoing blood loss, hemodynamic status, the presence of sepsis or consumption coagulopathy, or some concomitant drugs on efficacy outcomes were not taken into account.

In conclusion, this post-marketing study provides data on the safety of octaplas in critically ill pediatric patients who require replacement of multiple coagulation factors. The results of the present study and those of previous studies of octaplas support its use in this patient population.

## Data Availability Statement

The datasets presented in this study can be found in online repositories. The names of the repository/repositories and accession number(s) can be found at: ClinicalTrials.gov (NCT02050841).

## Ethics Statement

The studies involving human participants were reviewed and approved by Washington University St. Louis, Emory University, Western Institutional Review Boards, University of Minnesota. Written informed consent to participate in this study was provided by the participants' legal guardian/next of kin.

## Author Contributions

PS contributed to the study design, patient enrollment, data analysis, and preparation of the manuscript. SB contributed to patient enrollment, data collection and analysis, and preparation of the manuscript. JA contributed to study design, enrolled patients, and contributed to manuscript preparation. All authors contributed to the article and approved the submitted version.

## Conflict of Interest

PS is a consultant for Octapharma, Cerus and Entegrion. The remaining authors declare that the research was conducted in the absence of any commercial or financial relationships that could be construed as a potential conflict of interest.
